# Intravenous antibiotics at the index emergency department visit as an independent risk factor for hospital admission at the return visit within 72 hours

**DOI:** 10.1371/journal.pone.0264946

**Published:** 2022-03-18

**Authors:** Shao-Yung Lin, Chih-Wei Sung, Edward Pei-Chuan Huang, Chi-Hsin Chen, Cheng-Yi Fan, Hsin-Yu Lee, Chien-Tai Huang, Yu-Sheng Huang, Bo-Yu Zhuang, Cheng-Heng Liu, Jia-How Chang

**Affiliations:** 1 Department of Emergency Medicine, National Taiwan University Hospital, Taipei City, Taiwan; 2 Department of Emergency Medicine, National Taiwan University Hospital Hsin-Chu Branch, Hsinchu City, Taiwan; 3 Department of Family Medicine, New Taipei City Hospital, New Taipei City, Taiwan; 4 Department of Medical Education, National Taiwan University Hospital, Taipei City, Taiwan; National Taiwan University, TAIWAN

## Abstract

**Introduction:**

Although infection was the most common symptom in patients returning to the ED, whether intravenous antibiotic administration at the index visit could serve as an indicator of patients with infectious diseases at high risk for hospital admission after returning to the ED within a short period of time remains unclear. The study aimed to investigate the potential risk factors for hospital admission in patients returning to the ED within 72 hours with a final diagnosis of infectious diseases.

**Material and methods:**

This retrospective cohort study analyzed return visits to the ED from January to December 2019. Adult patients aged >20 years who had a return visit to the ED within 72 hours with an infectious disease were included herein. In total, 715 eligible patients were classified into the intravenous antibiotics and non-intravenous antibiotics group (reference group). The outcome studied was hospital admission to general ward and intensive care unit (ICU) at the return visits.

**Results:**

Patients receiving intravenous antibiotics at index visits had significantly higher risk—approximately two times—for hospital admission at the return visits than those did not (adjusted odds ratio = 2.47, 95% CI = 1.34–4.57, *p* = 0.004). For every 10 years increase in age, the likelihood for hospital admission increased by 38%. Other factors included abnormal respiratory rate and high C-reactive protein levels.

**Conclusions:**

Intravenous antibiotic administration at the index visit was an independent risk factor for hospital admission at return visits in patients with an infection disease. Physicians should consider carefully before discharging patients receiving intravenous antibiotics.

## Introduction

Unplanned return visits to the emergency departments (EDs) refer to the phenomenon wherein patients initially discharged from the ED unexpectedly revisit the ED within a short time, generally within 72 h. This may suggest higher risk for disease progression in these patients, greater economic burden on the health care system, and health care provider overload, especially in crowded EDs [1–4]. Previous studies have widely regarded unplanned return visits to the ED as an indicator of quality of care in the EDs [5], 3% to 32% of which have been found to be avoidable [6, 7]. However, some studies have proposed that hospital admission at return visits rather than the rates or frequencies of ED return visits themselves may imply potential deficiencies in management during the index visit, with the rate of post-return hospital admission being regarded as the outcome variable [5, 8, 9].

Infection remains the leading cause of ED visitations, as well as return visits within 72 h [10–12]. A study demonstrated that fever or infection-related complaints were the most common initial presentations among patients who had return visits to the ED [12]. Antibiotics, which can be prescribed either orally or intravenously, remains the gold standard treatment for patients with suspected or confirmed infection. Physicians generally administer intravenous antibiotics to patients with potentially severe infections, comorbidities, or specific types of infection, with hospital admission being highly indicated. The utilization of intravenous antibiotics has also been one of the indications for hospital admission, which implies a longer treatment course in recipient patients. However, previous studies have demonstrated inconsistent results on the association between intravenous antibiotics and the risk of poor outcomes in patients with unplanned ED return visits [13, 14].

The potential for intravenous antibiotic administration to serve as an indicator of patients at high risk or hospital admission after returning to EDs is certainly intriguing. However, published literature exploring such an issue remains insufficient. Whether the administer of intravenous antibiotics in patients with potential infectious diseases on hospital admission if they had an unplanned return to ED was unclear. The current study therefore investigated the association between intravenous antibiotic administration at the index ED visit and the risk of hospital admission at the return visit.

## Material and methods

### Ethics statement

The study was approved by Institution Review Board of National Taiwan University Hsin-Chu Hospital (no. 109-003-E) and was conducted in accordance with Declaration of Helsinki and ICH-GCP guidelines. The approving body waived the need for written informed consent given the retrospective nature of this study with minimal intervention.

### Study design and setting

This single-center, retrospective cohort study was conduct at National Taiwan University Hsin-Chu Hospital, with a capacity of 829 beds and more than 1700 staff. On average, over 60,000 patients visit the ED annually. Patients were enrolled from January 2019 through December 2019, after which analysis was performed from July 2020 through November 2020.

### Participants and interventions

Patients who satisfied the following criteria were included: aged over 20 years old, unplanned ED return visits after an index visit, underwent standard evaluation in the ED and with a final diagnosis of an infectious disease at return visits. In contrast, those who eloped from the ED during their visit, refused access to any of their medical records, or had missing data from their charts were ultimately excluded from analysis. An unplanned return visit was defined as any return visit to the ED within 72 h after being discharge therefrom.

Enrolled patients were divided into two groups according to the use of intravenous antibiotics during the index ED admission. Intravenous antibiotic administration was confirmed when antibiotic agents were administered via an intravenous catheter. To identify the effects of intravenous antibiotics on outcomes, patients were subsequently classified into the exposure (with intravenous antibiotics) and non-exposure (without intravenous antibiotics) groups.

### Measurements

Medical records were examined for information regarding age, sex, vital signs, triage level, pre-existing comorbidities, symptoms, laboratory data, diagnosis, and disposition. Vital signs, including body temperature, respiratory rate, pulse rate, and blood pressure, were recorded upon triage. Our hospital used a 5-level triage system called the Taiwan Triage and Acuity Scale (TTAS) computerized system that was modified from the five-level Canadian Triage and Acuity Scale implemented nationally since 2010 [15]. Afebrile patients were defined as those with a body temperature of less than 38°C upon triage at the index visit. Pre-existing comorbidities included significant systematic disease, like hypertension, diabetes mellitus, coronary artery disease, chronic kidney disease, malignancy, and chronic obstruction pulmonary disease. Common symptoms, such as headache, chest pain, dyspnea, abdominal pain, vomiting dysuria, were also included. Laboratory data extracted white blood cell (WBC) count, neutrophilic granulocyte percentage, and levels of hemoglobin, C-Reactive protein, sodium, potassium, creatinine, and alanine amino transferase. An attending physician established the final diagnosis, which was classified into four categories, namely respiratory, circulation, gastrointestinal, and infection, at the end of the return visits. Variables determined from the medical charts were recorded by independent research assistants and physicians, with ambiguous records being rechecked and decided on by another senior physician.

### Outcomes

The overall follow-up duration for each patient was from the index ED visit until the discharge after the return visit. The outcome was ward admission or intensive care unit (ICU) admission during the return visits.

### Statistical analysis

A data analyst blinded to study design and manuscript draft was in charge of data collection and pre-processing. Normality of the variables was determined using the Shapiro-Wilk test [16]. Continuous variables were presented as mean ± standard deviation and were compared using the independent t-test when variables satisfied the normality criteria. Categorical data were presented as numbers with percentages and were compared using the chi-squared test.

Risk for admission to general ward and ICU between the exposure and non-exposure groups were compared using univariate logistic regression analysis. Potential confounders were adjusted using a multivariate logistic regression model. Odds ratios (ORs) with 95% confidence intervals (CIs) were also presented. Age was grouped by decile of 10-year change, pulse rate was classified into normal and abnormal groups, the normal range in which was 60–100 beats per minute. Respiratory rate was also classified into normal and abnormal groups, in which the normal rate was 12–21 breaths per minute. All statistical analyses were performed using SAS version 9.4 (SAS Institute, Cary, NC), with a two-sided *P* value of < 0.05 indicating statistical significance.

## Results

### Patient enrollment

**Fig 1:**
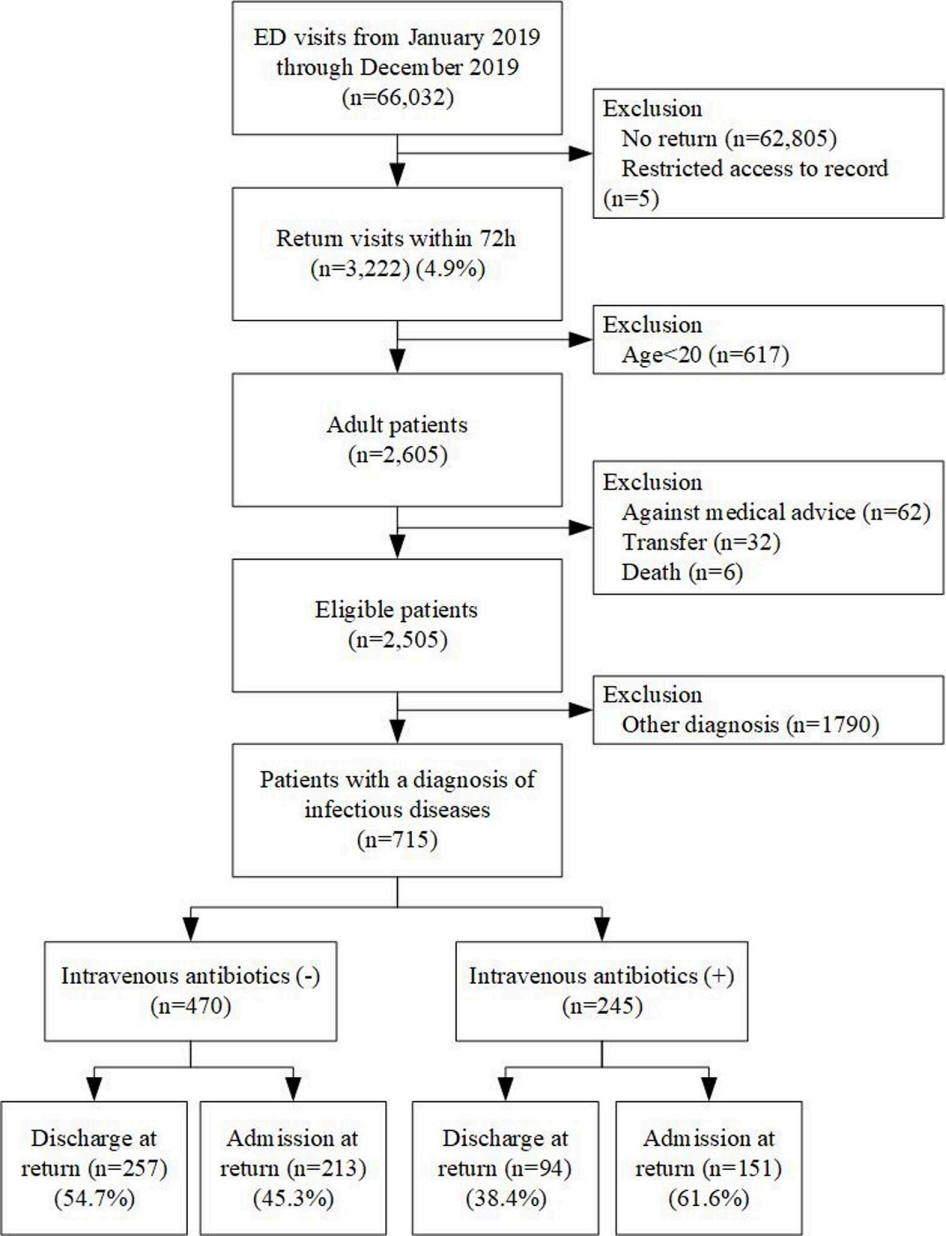
Study flow and patient recruitment.

Initially, more than 66,000 patients visited the ED in 2019. Among them, 62,805 patients were excluded because they did not have a return visit to the ED, and 5 patients were excluded because of the inability to access their medical records. In total, 3,222 (approximately 5%) patients returned to ED within 72 hours. Non-adults (617 patients) were excluded as per inclusion criteria. Subsequently, 100 patients were excluded because they were discharged against medical advice (n = 62), due to hospital transfers (n = 32) and deaths (n = 6). Finally, patients without a final diagnosis of infectious diseases (n = 1790) were excluded. The included patients (n = 715) were classified into intravenous antibiotic (n = 245) and non-intravenous antibiotic (n = 470) groups. In the non-intravenous antibiotic group, half of the patients were discharged after their returning to ED within 72 hours. In the intravenous antibiotic group, more than 60% patients required hospital admission (Fig 1).

### Patient characteristics at the index visit

Table 1 compares non-intravenous antibiotic and intravenous antibiotic groups in demographics, pre-existing comorbidities, vital signs, triage information, symptoms, and laboratory data. Generally, enrolled patients had an average age 58 years old, with males being the predominant sex. Hypertension, diabetes mellitus, and malignancy were leading pre-existing diseases, followed by coronary artery disease and chronic kidney disease. In addition to chronic kidney disease, no significant differences were noted between both groups.

**Table 1:** Comparison of demographics, pre-comorbidities, vitals, symptoms, lab data at index visit in patients with a diagnosis of infectious cohort (infection cohort).

Variables	Infection cohort (n = 715)	Without IV_Abx (n = 370)	With IV_Abx (n = 245)	*p*
**Age (years)**	58.0 ± 20.5	56.9 ± 20.7	60.0 ± 20.0	0.057
**Male (%)**	384 (53.7)	254 (54.0)	130 (53.1)	0.803
**Pre-comorbidities**				
Hypertension	259 (36.2)	161 (34.3)	98 (40.0)	0.129
Diabetes mellitus	171 (23.9)	102 (21.7)	69 (28.2)	0.055
Coronary artery disease	81 (11.3)	50 (10.6)	31 (12.7)	0.420
Chronic kidney disease	57 (9.0)	30 (6.4)	27 (11.0)	0.030
Malignancy	115 (16.1)	70 (14.9)	45 (18.4)	0.230
COPD	41 (5.7)	27 (5.7)	14 (5.7)	0.987
**Vitals**				
SBP (mmHg)	145.5 ± 29.7	146.7 ± 29.8	143.2 ± 29.5	0.141
DBP (mmHg)	78.7 ± 15.7	79.0 ± 15.8	78.1 ± 15.5	0.477
Body temperature	37.5 ± 1.0	37.3 ± 1.0	37.7 ± 1.1	<0.001
Pulse rate (bpm)	98.8 ± 19.7	98.2 ± 20.2	99.9 ± 19.0	<0.001
Respiratory rate	20.3 ± 2.0	20.3 ± 2.0	20.5 ± 2.0	0.289
**Triage level**				0.214
1 or 2	98 (13.7)	59 (12.6)	39 (15.9)	
3 or 4 or 5	617 (86.3)	411 (87.5)	206 (84.1)	
**Symptoms**				
Headache	56 (7.8)	47 (10.0)	9 (3.7)	0.003
Chest pain	36 (5.0)	27 (5.7)	9 (3.7)	0.229
Weakness	62 (8.7)	36 (7.7)	26 (10.6)	0.183
Dyspnea	53 (7.4)	29 (6.2)	24 (9.8)	0.079
Cough	149 (20.8)	98 (20.9)	51 (20.8)	0.991
Abdominal pain	166 (23.2)	115 (24.5)	51 (20.8)	0.272
Vomiting	72 (10.1)	53 (11.3)	19 (7.8)	0.417
Diarrhea	76 (6.8)	58 (12.3)	18 (7.4)	0.040
Flank pain	24 (3.4)	16 (3.4)	8 (3.3)	0.922
Dysuria	34 (4.8)	18 (3.8)	16 (6.5)	0.107
Urinary frequency	25 (3.5)	11 (2.3)	14 (5.7)	0.020
Chills	130 (18.2)	63 (13.4)	67 (27.4)	<0.001
Soreness	92 (12.9)	72 (15.3)	20 (8.2)	0.007
Edema	39 (5.5)	17 (3.6)	22 (9.0)	0.003
**Lab**				
WBC	10.2 ± 4.4	9.9 ± 4.0	10.5 ± 4.8	0.127
Seg (%)	78.3 ± 12.0	78.0 ± 11.9	78.6 ± 12.1	0.612
Hb	12.9 ± 2.3	13.0 ± 2.3	12.7 ± 2.2	0.212
CRP	4.3 ± 5.1	3.5 ± 4.8	5.0 ± 5.3	0.014
Na	134.1 ± 3.8	134.1 ± 4.1	134.0 ± 3.5	0.854
K	3.9 ± 0.7	3.8 ± 0.6	3.9 ± 0.7	0.366
Cre	1.3 ± 1.5	1.2 ± 1.3	1.4 ± 1.8	0.145
ALT	30.9 ± 53.4	29.2 ± 41.0	33.0 ± 65.9	0.473

COPD = chronic obstructive pulmonary disease; CRP = C-reactive protein; DBP = diastolic blood pressure; Hb = hemoglobin; IV_Abx = intravenous antibiotics; SBP = systolic blood pressure; WBC = white blood cell.

Patients in the intravenous antibiotic group had significantly higher body temperature and pulse rate compared to those in the non-intravenous antibiotic group. Around one-seventh of the patients were triaged as level 1 or 2, and there was no significant intergroup difference. At the index visit, abdominal pain was the most common symptom, affecting 23.2% of all patients. Coughing, chills, soreness and vomiting accounted for more than 10% of the symptoms. Patients who received intravenous antibiotics at the index visit had higher rates of headache, diarrhea, dysuria, urinary frequency (i.e., the need to urinate multiple times), chills, and edema but lower rates of headache and chest pain compared to those who did not. Moreover, those who received intravenous antibiotics had a significantly higher CRP level.

### Patient characteristics of the return visits

The characteristics of patients who had the unplanned return visits are detailed in Table 2. Accordingly, nearly half of the patients returned to hospital within the first day after discharge. Nearly two times higher proportion of diarrhea in the non-intravenous antibiotic group than that in the intravenous antibiotic group. Also, a higher rate of cough and headache was noted in the non-intravenous antibiotic group than that in the intravenous antibiotic group.

**Table 2:** Comparison of timing of return visit, vitals, symptoms, lab data, diagnosis, and outcome at return visit in the infection cohort.

Variables	Infection cohort (n = 715)	Without IV_Abx (n = 470)	With IV_Abx (n = 245)	*p*
**ED returns**				0.019
<24 h	348 (48.7)	217 (46.2)	131 (53.5)	
24 h to 48 h	234 (32.7)	152 (32.3)	82 (33.5)	
48 h to 72 h	133 (18.6)	101 (21.5)	32 (13.0)	
**Vitals**				
SBP (mmHg)	140.2 ± 27.9	140.5 ± 28.6	139.7 ± 26.5	0.712
DBP (mmHg)	76.2 ± 15.1	76.3 ± 14.8	76.0 ± 15.7	0.729
Body temperature	37.4 ± 1.1	37.5 ± 1.1	37.3 ± 1.1	0.021
Pulse rate (bpm)	97.1 ± 19.5	98.3 ± 19.4	95.0 ± 19.6	0.034
Respiratory rate	20.4 ± 2.3	20.3 ± 2.2	20.4 ± 2.4	0.434
**Triage level**				0.697
1 or 2	137 (19.2)	92 (19.6)	45 (18.4)	
3 or 4 or 5	578 (80.8)	378 (80.4)	200 (81.6)	
**Symptoms**				
Headache	37 (5.2)	30 (6.4)	7 (2.9)	0.043
Chest pain	31 (4.3)	25 (5.3)	6 (2.5)	0.074
Weakness	54 (7.6)	39 (8.3)	15 (6.1)	0.296
Dyspnea	70 (9.8)	48 (10.2)	22 (9.0)	0.600
Cough	112 (15.7)	83 (17.7)	29 (11.8)	0.042
Abdominal pain	142 (19.9)	99 (21.1)	43 (17.6)	0.264
Vomiting	86 (12.0)	61 (13.0)	25 (10.2)	0.279
Diarrhea	74 (10.4)	58 (12.3)	16 (6.5)	0.016
Flank pain	29 (4.1)	19 (4.0)	10 (4.1)	0.980
Dysuria	23 (3.2)	14 (3.0)	9 (3.7)	0.617
Urinary frequency	17 (2.4)	11 (2.3)	6 (2.5)	0.928
Chills	125 (17.5)	79 (16.8)	46 (18.8)	0.511
Soreness	57 (8.0)	43 (9.2)	14 (5.7)	0.108
Edema	50 (7.0)	27 (5.7)	23 (9.4)	0.070
**Lab**				
WBC	10.3 ± 4.8	10.4 ± 4.9	10.0 ± 4.6	0.325
Seg (%)	77.6 ± 12.6	77.9 ± 12.2	76.8 ± 13.5	0.396
Hb	12.6 ± 2.3	12.6 ± 2.3	12.2 ± 2.3	0.002
CRP	8.1 ± 8.3	7.7 ± 8.0	9.0 ± 9.0	0.275
Na	133.9 ± 4.2	133.8 ± 4.3	134.1 ± 4.0	0.499
K	3.8 ± 0.6	3.8 ± 0.6	3.8 ± 0.7	0.721
Cre	1.4 ± 1.8	1.4 ± 1.5	1.7 ± 2.3	0.141
ALT	39.3 ± 92.3	37.8 ± 95.6	43.8 ± 81.4	0.630
**Outcome**				
Hospital admission	364 (50.9)	213 (45.3)	151 (61.6)	<0.001

COPD = chronic obstructive pulmonary disease; CRP = C-reactive protein; DBP = diastolic blood pressure; Hb = hemoglobin; IV_Abx = intravenous antibiotics; SBP = systolic blood pressure; WBC = white blood cell.

Almost 51% patients were admitted to general hospital wards or ICU. Among those who received intravenous antibiotics at the index visit, 61.6% were admitted to hospital. A higher proportion of hospital admission in intravenous antibiotic group than that in non-intravenous antibiotic group (61.6% vs. 45.3%, *p* < 0.001).

### Association between intravenous antibiotics at the index visits and hospital admission at the return visits

Table 3 demonstrates the association between potential factors at the index visit and hospital admission at the return visit. In the infection cohort, after adjusting for age, sex, symptoms, vital signs, and lab data, the results indicated that patients who received intravenous antibiotics at their index visits had significantly higher likelihood—more than two-fold—for hospital admission at their return visit than those who did not (adjusted OR [aOR] = 2.47, 95% CI = 1.34–4.57, *p* = 0.004). Age was also identified as a risk factor for admission at the return visit, with a higher likelihood in hospital admission as age increased. Patients had a 38% higher risk for hospital admission at their return visit with every 10-year increase in age (OR = 1.38, 95% CI = 1.17–1.63, *p* < 0.001). Patients with an abnormal respiratory rate exhibited significantly higher probability for admission (aOR = 2.96, 95% CI = 1.20–7.28, *p* = 0.018). Patients with increased CRP levels had approximately 9% higher likelihood for hospital admission after their return visit to the ED. Patients presenting with cough were less likely to be admitted during return visits to the ED; however, no other significant association was observed between the symptoms and hospital admission during return visits to the ED.

**Table 3:** The factors on hospital or ICU admission after ED return visits in the infection cohort.

	Adjusted OR (95% CI)
**Age (per 10 year)**	1.38 (1.17–1.63)
**Male**	1.49 (0.83–2.68)
**IV_Abx**	2.47 (1.34–4.57)
**Vitals**	
Fever	1.23 (0.64–2.37)
Pulse rate	1.54 (0.81–2.92)
Respiratory rate	2.96 (1.20–7.28)
SBP	0.99 (0.97–1.00)
DBP	1.01 (0.98–1.04)
**Symptoms**	
Headache	1.93 (0.55–4.81)
Chest pain	0.38 (0.09–1.53)
Weakness	1.10 (0.42–2.85)
Dyspnea	0.70 (0.21–2.35)
Cough	0.26 (0.11–0.60)
Dysuria	7.07 (0.75–66.42)
Urinary frequency	0.31 (0.03–3.06)
Chills	0.62 (0.30–1.30)
Edema	0.53 (0.17–1.64)
**Lab**	
WBC	1.06 (0.98–1.13)
CRP	1.09 (1.02–1.17)

CI = confidence interval; CRP = C-reactive protein; DBP = diastolic blood pressure; ED = emergency department; Hb = hemoglobin; ICU = intensive care unit; IV_Abx = intravenous antibiotics; NA = not available; OR = odds ratio; SBP = systolic blood pressure; WBC = white blood cell.

Table 4 shows the use of the intravenous antibiotics on different symptoms. Patients who had the symptoms of abdominal pain (12.4%) received the most intravenous antibiotics, followed by symptoms of chills (10.5%).

**Table 4:** Comparison of symptoms and use of intravenous antibiotics at the index visit.

Symptoms	N	Without IV_Abx	With IV_Abx
Headache	158	144 (20.1)	14 (2.0)
Chest pain	209	193 (27.0)	16 (2.2)
Weakness	203	164 (22.9)	39 (5.5)
Dyspnea	213	169 (23.6)	44 (6.2)
Cough	278	208 (29.1)	70 (9.8)
Abdominal pain	582	493 (69.0)	89 (12.4)
Vomiting	290	252 (35.2)	38 (5.3)
Diarrhea	169	139 (19.4)	30 (4.2)
Flank pain	105	88 (12.3)	17 (2.4)
Dysuria	66	48 (6.7)	18 (2.5)
Urinary frequency	48	29 (4.1)	19 (2.7)
Chills	165	90 (12.6)	75 (10.5)
Soreness	177	146 (20.4)	31 (4.3)
Edema	116	86 (12.0)	30 (4.2)

IV_Abx = intravenous antibiotics.

## Discussion

The current study demonstrated that multiple factors affect the increased risk for hospital admission among patients who visited the ED again and had a final diagnosis of infectious diseases. These factors included age, respiratory rate, evaluated CRP levels and, most importantly, intravenous antibiotic administration at their index visits. Compared to patients who did not receive intravenous antibiotics at the index visit, those who did but were subsequently discharged had higher risk for post-return hospital admission. As such, intravenous antibiotic administration was herein identified as a significant independent risk factor for hospital admission in the infection cohort. The strength of the present study lies in its use of precise data collected from medical records, which were subsequently reviewed by physicians, rather than utilizing integrated databases that contain incorrect or inappropriate records. Considering that the physicians were engaged in reviewing the medical records, the symptoms, diagnosis and other variables in the study can be considered more precise; therefore, information bias can be minimized [10, 17]. Additionally, the current study included approximately 30 symptoms collected from narratives detailed in the medical records of patients with unplanned return visits to the ED.

Some implications regarding the association between intravenous antibiotic administration at the index visit and hospital admission at return visits are described below. First, for ED physicians, the decision making in administering intravenous antibiotics on the basis of symptoms, physical examination, and laboratory data were timely and accurate. Accordingly, the administration of intravenous antibiotics itself implies a potentially serious infection or deteriorated condition in the near future. Regardless of the reasons for being discharged at the index visit, these patients who were discharged had a higher probability for hospital admission when they returned to the ED in a short time. This may imply that patients receiving intravenous antibiotics should be admitted at their index ED visit as possible, rather than being discharged. It may also decrease the rate of hospital admission upon returning to the ED. Second, to admit the potentially ill patients who would have ED return visit earlier at their index visit may decrease complication rate because these patients received definite treatment in an earlier time. Third, if the patients were admitted, the fewer return visits were noted so that it may resolve the overcrowded EDs, and decrease medical cost. Fourth, intravenous antibiotics themselves were intended for severe infections and were reported as one of the indications for hospital admission. In 2018, Jorgeson et al. identified intravenous antibiotics as a risk factor for early ED returns among patients with urinary tract infection [13]. The study further indicated that intravenous antibiotic administration was an independent risk factor for subsequent hospital admission among those with not only urinary tract infection but also other potential infectious diseases. Another study also promoted the early model of hospital admission at the index ED visit if the patients were indicated [18].

Notably, symptoms were not associated with increased risk of hospital admission. One potential explanation may originate from small sample size. Although <4% of the patients presented with dysuria, they exhibited extremely high risk for hospital admission despite not reaching statistical significance. On the other hand, the severity of infection or inflammatory reaction is more likely to be explained by the vital signs rather than the symptoms. Interestingly, patients presenting with cough had a lower likelihood to be admitted. In other words, the presence of cough in patients with upper respiratory infection may not reflect the severity of infection. Furthermore, some symptoms may be associated with vital signs, such as dyspnea and respiratory rate. Although respiratory rate was associated with higher likelihood admission, dyspnea was not. Such phenomena can be explained by that the presentation of dyspnea was a subjective complaint which did not require the patients to be actually tachypneic. For example, patients with anxiety often present with a symptom of dyspnea.

Other factors associated with higher risk of hospital admission at return visits included age and CRP levels. Elderly individuals generally often suffer from multiple comorbidities and have higher risk for hospital admission at return visits, potentially leading to poor outcomes [12, 19]. Likewise, CRP levels have been traditionally and widely considered as indicators of infection severity [20].

We further analyzed the non-infection cohort and overall cohort (combined with infection and non-infection cohorts). In the overall cohort, the characteristics at the index and the return visit, as shown in S1 and S2 Tables, showed similar and consistent results with the infection cohort (Tables 1 and 2) including the distribution of age, male gender, pre-comorbidities and triage levels. However, the rate of chills, dysuria and cough seemed to be remarkably higher in the infection cohort. The mean serum levels of WBC and CRP were also higher in the infection cohort. It was not surprising that these symptoms and lab panels may reflect the potential infection disease and its severity. Additionally, S3 and S4 Tables demonstrated the characteristics at the index and the return visit in non-infection cohort. Although intravenous antibiotics at the index visit in the non-infection cohort was not significantly associated with a higher probability of hospital admission, still, 7% of those patients received intravenous antibiotics at their index visits to ED. One potential explanation is overtreatment. Some physicians may administer intravenous antibiotics aggressively for patients suspicious of infection or more vulnerable to infection but actually they did not have an infection. In this condition, intravenous antibiotics may not serve as an indicator of infection disease or even its severity. It may reflect the weak association of intravenous antibiotics and risk of hospital admission in non-infection cohort.

### Limitations

Some limitations of the current study are worth noting. First, the current data were collected from a single hospital in 2019, which may affect external generalizability. As such, further investigations in diverse populations may be needed to validate our results. Second, although all variables collected from the medical records were carefully reviewed by several physicians, which minimized information error, missing data for blood work and vital signs may cause misinterpretation. Third, some vitals were missing that may influence the result. We listed the missing number in S5 Table. After the rechecking data, less than 2% vitals were missing. Fourth, in spite of some potential recording errors by manual recording, we carefully examine the data, and by rechecking the data randomly to minimize this effect. Lastly, special scenarios, including patients with substance disorder, those with unstable housing, or with certain mental health conditions such as anxiety, may influence the effects of intravenous antibiotics on outcomes given that they inflate ED return visit rates despite not needing admission.

## Conclusions

Multiple factors were associated with hospital admission at the return visit to the ED, among which intravenous antibiotic administration was identified as an independent risk factor. Intravenous antibiotics at the index visit may reflect an increased risk for hospital admission in patients with a diagnosis of infectious diseases. Physicians should therefore carefully review patients prescribed intravenous antibiotics, regardless of febrile status, and should not discharge patients with suspected infectious diseases.

## Supporting information

S1 Table Comparison of demographics, pre-comorbidities, vitals, symptoms, lab data at index visit in overall cohort. (DOCX)

S2 Table Comparison of timing of return visit, vitals, symptoms, lab data, diagnosis, and outcome at return visit in overall cohort. (DOCX)

S3 Table Comparison of demographics, pre-comorbidities, vitals, symptoms, lab data at index visit in the non-infection cohort. (DOCX)

S4 Table Comparison of timing of return visit, vitals, symptoms, lab data, diagnosis, and outcome at return visit in the non-infection cohort. (DOCX)

S5 Table Missing values in vitals. (DOCX)
