# Supplementary material for: Intravenous antibiotics at the index emergency department visit as an independent risk factor for hospital admission at the return visit within 72 hours
Source: PLoS One. 2022 Mar 18;17(3):e0264946. doi: 10.1371/journal.pone.0264946 (PMC8932564; doi:10.1371/journal.pone.0264946)
Supplement: S1 Table — (DOCX) [file pone.0264946.s001.docx]

| S1 Table. Comparison of demographics, pre-comorbidities, vitals, symptoms, lab data at index visit in overall cohort | | | | |
| --- | --- | --- | --- | --- |
| Variables | Total (n=2,505) | Without IV_Abx (n=2,137) | With IV_Abx  (n=368) | *p* |
| **Age (years)** | 59.8 ± 19.7 | 59.6 ± 19.7 | 60.9 ± 20.0 | 0.274 |
| **Male (%)** | 1320 (52.7) | 1122 (52.5) | 198 (53.8) | 0.644 |
| **Pre-comorbidities** |  |  |  |  |
| Hypertension | 972 (38.8) | 823 (38.5) | 149 (40.5) | 0.472 |
| Diabetes mellitus | 590 (23.6) | 497 (23.3) | 92 (25.3) | 0.400 |
| Coronary artery disease | 365 (14.6) | 312 (14.6) | 53 (14.4) | 0.921 |
| Chronic kidney disease | 236 (9.4) | 290 (8.9) | 46 (12.5) | 0.029 |
| Malignancy | 399 (15.9) | 330 (15.4) | 69 (18.8) | 0.109 |
| COPD | 107 (4.3) | 84 (3.9) | 23 (6.3) | 0.042 |
| **Vital signs** |  |  |  |  |
| SBP (mmHg) | 148.8 ± 32.0 | 149.6 ± 32.0 | 144.3 ± 31.6 | 0.004 |
| DBP (mmHg) | 80.4 ± 16.1 | 80.8 ± 16.1 | 78.4 ± 15.8 | 0.008 |
| Body temperature | 36.9 ± 0.9 | 36.8 ± 0.8 | 37.5 ± 1.1 | <0.001 |
| Pulse rate (bpm) | 92.1 ± 19.7 | 91.0 ± 19.6 | 98.2 ± 19.1 | <0.001 |
| Respiratory rate | 20.2 ± 2.0 | 20.2 ± 1.9 | 20.6 ± 2.5 | 0.003 |
| **Triage** |  |  |  | 0.032 |
| 1 or 2 | 359 (14.3) | 293 (13.7) | 66 (17.9) |  |
| 3 or 4 or 5 | 2146 (85.7) | 1844 (86.3) | 302 (82.1) |  |
| **Symptoms** |  |  |  |  |
| Headache | 158 (6.3) | 144 (6.7) | 14 (3.8) | 0.003 |
| Chest pain | 209 (8.3) | 193 (9.0) | 16 (4.4) | 0.003 |
| Weakness | 203 (8.1) | 164 (7.7) | 39 (10.6) | 0.058 |
| Dyspnea | 213 (8.5) | 169 (7.9) | 44 (12.0) | 0.010 |
| Cough | 278 (11.1) | 208 (9.7) | 70 (19.0) | <0.001 |
| Abdominal pain | 582 (23.2) | 493 (23.1) | 89 (24.2) | 0.640 |
| Vomiting | 290 (11.6) | 252 (11.8) | 38 (10.3) | 0.417 |
| Diarrhea | 169 (6.8) | 139 (6.5) | 30 (8.2) | 0.244 |
| Flank pain | 105 (4.2) | 88 (4.1) | 17 (4.6) | 0.657 |
| Dysuria | 66 (2.6) | 48 (2.3) | 18 (4.9) | 0.003 |
| Urinary frequency | 48 (1.9) | 29 (1.4) | 19 (5.2) | <0.001 |
| Chills | 165 (6.6) | 90 (4.2) | 75 (20.4) | <0.001 |
| Soreness | 177 (7.1) | 146 (6.8) | 31 (8.4) | 0.271 |
| Edema | 116 (4.6) | 86 (4.2) | 30 (8.2) | <0.001 |
| **Lab** |  |  |  |  |
| WBC | 9.4 ± 5.2 | 8.9 ± 3.5 | 10.9 ± 8.5 | <0.001 |
| Seg (%) | 74.2 ± 12.5 | 73.0 ± 12.5 | 77.8 ± 12.0 | <0.001 |
| Hb | 12.8 ± 2.5 | 12.8 ± 2.5 | 12.6 ± 2.3 | 0.191 |
| CRP | 3.3 ± 4.6 | 2.5 ± 4.0 | 4.5 ± 5.2 | <0.001 |
| Na | 134.4 ± 4.3 | 134.4 ± 4.4 | 134.2 ± 3.7 | 0.335 |
| K | 3.9 ± 0.7 | 3.9 ± 0.6 | 3.9 ± 0.7 | 0.592 |
| Cre | 1.5 ± 1.9 | 1.5 ± 1.8 | 1.5 ± 1.9 | 0.681 |
| ALT | 33.9 ± 116.1 | 34.3 ± 128.0 | 32.8 ± 61.6 | 0.850 |
| COPD = chronic obstructive pulmonary disease; CRP = C-reactive protein; DBP = diastolic blood pressure; Hb = hemoglobin; IV_Abx = intravenous antibiotic; SBP = systolic blood pressure; WBC = white blood cell | | | | |
