# Supplementary material for: Intravenous antibiotics at the index emergency department visit as an independent risk factor for hospital admission at the return visit within 72 hours
Source: PLoS One. 2022 Mar 18;17(3):e0264946. doi: 10.1371/journal.pone.0264946 (PMC8932564; doi:10.1371/journal.pone.0264946)
Supplement: S2 Table — (DOCX) [file pone.0264946.s002.docx]

| S2 Table. Comparison of timing of return visit, vitals, symptoms, lab data, diagnosis, and outcome at return visit in overall cohort | | | | |
| --- | --- | --- | --- | --- |
| Variables | Total (n=2,505) | Without IV_Abx (n=2,137) | With IV_Abx  (n=368) | *p* |
| **ED returns** |  |  |  | 0.166 |
| <24 h | 1151 (46.0) | 968 (45.3) | 183 (49.7) |  |
| 24 h to 48 h | 822 (32.8) | 703 (32.9) | 119 (32.3) |  |
| 48 h to 72 h | 532 (21.2) | 466 (21.8) | 66 (17.9) |  |
| **Vital signs** |  |  |  |  |
| SBP (mmHg) | 144.9 ± 30.7 | 145.6 ± 31.1 | 140.1 ± 27.6 | 0.001 |
| DBP (mmHg) | 78.6 ± 15.7 | 78.9 ± 15.7 | 76.6 ± 15.8 | 0.009 |
| Body temperature | 36.9 ± 0.9 | 36.9 ± 0.8 | 37.2 ± 1.0 | <0.001 |
| Pulse rate (bpm) | 91.0 ± 19.4 | 90.4 ± 19.3 | 93.8 ± 19.2 | 0.002 |
| Respiratory rate | 20.3 ± 2.3 | 20.3 ± 2.2 | 20.4 ± 2.5 | 0.359 |
| **Triage** |  |  |  | 0.339 |
| 1 or 2 | 446 (17.8) | 374 (17.5) | 72 (19.6) |  |
| 3 or 4 or 5 | 2059 (82.2) | 1763 (82.5) | 296 (80.4) |  |
| **Symptoms** |  |  |  |  |
| Headache | 140 (5.6) | 128 (6.0) | 12 (3.3) | 0.035 |
| Chest pain | 186 (7.4) | 172 (8.1) | 14 (3.8) | 0.004 |
| Weakness | 201 (8.0) | 181 (8.5) | 20 (5.4) | 0.032 |
| Dyspnea | 244 (9.7) | 200 (9.4) | 44 (12.0) | 0.121 |
| Cough | 181 (7.2) | 145 (6.8) | 36 (9.8) | 0.040 |
| Abdominal pain | 532 (21.2) | 458 (21.4) | 74 (20.1) | 0.567 |
| Vomiting | 296 (11.8) | 258 (12.1) | 38 (10.3) | 0.338 |
| Diarrhea | 143 (5.7) | 117 (5.5) | 26 (7.1) | 0.225 |
| Flank pain | 105 (4.2) | 88 (4.1) | 17 (4.6) | 0.657 |
| Dysuria | 37 (1.5) | 27 (1.3) | 10 (2.7) | 0.032 |
| Urinary frequency | 24 (1.0) | 17 (0.8) | 7 (1.9) | 0.044 |
| Chills | 144 (5.8) | 96 (4.5) | 48 (13.0) | <0.001 |
| Soreness | 113 (4.5) | 96 (4.5) | 17 (4.6) | 0.914 |
| Edema | 124 (5.0) | 94 (4.4) | 30 (8.2) | 0.002 |
| **Lab** |  |  |  |  |
| WBC | 9.5 ± 4.7 | 9.4 ± 4.7 | 9.9 ± 4.9 | 0.136 |
| Seg (%) | 75.1 ± 12.4 | 74.8 ± 12.2 | 76.6 ± 13.1 | 0.078 |
| Hb | 12.5 ± 2.4 | 12.6 ± 2.5 | 12.1 ± 2.4 | 0.004 |
| CRP | 5.8 ± 7.6 | 5.1 ± 7.0 | 8.4 ± 9.0 | 0.001 |
| Na | 134.1 ± 4.9 | 134.1 ± 5.0 | 134.0 ± 4.5 | 0.961 |
| K | 3.9 ± 1.2 | 4.0 ± 1.3 | 3.8 ± 0.7 | 0.170 |
| Cre | 1.6 ± 2.0 | 1.6 ± 2.0 | 1.6 ± 2.0 | 0.831 |
| ALT | 43.2 ± 140.3 | 42.7 ± 146.2 | 46.2 ± 89.3 | 0.806 |
| **Diagnosis** |  |  |  |  |
| Infection | 715 (28.5) | 470 (22.0) | 245 (66.6) | <0.001 |
| Circulation | 258 (10.3) | 246 (11.5) | 12 (3.3) | <0.001 |
| Respiratory | 257 (10.3) | 208 (9.7) | 49 (13.3) | 0.037 |
| Gastrointestinal | 651 (26.0) | 568 (26.6) | 83 (22.6) | 0.104 |
| **Outcome** |  |  |  |  |
| Hospital admission | 692 (27.6) | 504 (23.6) | 188 (51.1) | <0.001 |
| COPD = chronic obstructive pulmonary disease; CRP = C-reactive protein; DBP = diastolic blood pressure; Hb = hemoglobin; ICU = intensive care unit; IV_Abx = intravenous antibiotic; SBP = systolic blood pressure; WBC = white blood cell | | | | |
