# Supplementary material for: Intravenous antibiotics at the index emergency department visit as an independent risk factor for hospital admission at the return visit within 72 hours
Source: PLoS One. 2022 Mar 18;17(3):e0264946. doi: 10.1371/journal.pone.0264946 (PMC8932564; doi:10.1371/journal.pone.0264946)
Supplement: S3 Table — (DOCX) [file pone.0264946.s003.docx]

| S3 Table. Comparison of demographics, pre-comorbidities, vitals, symptoms, lab data at index visit in the non-infection cohort | | | | |
| --- | --- | --- | --- | --- |
| Variables | Total (n=1,790) | Without IV_Abx (n=1,667) | With IV_Abx  (n=123) | *p* |
| **Age (years)** | 60.5 ± 19.4 | 60.4 ± 19.3 | 62.5 ± 20.1 | 0.238 |
| **Male (%)** | 936 (52.3) | 868 (52.1) | 68 (55.3) | 0.491 |
| **Pre-comorbidities** |  |  |  |  |
| Hypertension | 713 (39.8) | 662 (39.7) | 51 (41.5) | 0.702 |
| Diabetes mellitus | 419 (23.4) | 395 (23.7) | 24 (19.5) | 0.290 |
| Coronary artery disease | 284 (15.9) | 262 (15.7) | 22 (17.9) | 0.525 |
| Chronic kidney disease | 179 (10.0) | 160 (9.6) | 19 (15.5) | 0.037 |
| Malignancy | 284 (15.9) | 260 (15.6) | 24 (19.5) | 0.251 |
| COPD | 66 (3.7) | 57 (3.4) | 9 (7.3) | 0.027 |
| **Vital signs** |  |  |  |  |
| SBP (mmHg) | 150.1 ± 32.8 | 150.4 ± 32.5 | 146.5 ± 35.5 | 0.204 |
| DBP (mmHg) | 81.1 ± 16.2 | 81.3 ± 16.2 | 78.9 ± 16.5 | 0.123 |
| Body temperature | 36.7 ± 0.6 | 36.7 ± 0.6 | 37.1 ± 0.9 | <0.001 |
| Pulse rate (bpm) | 89.3 ± 19.0 | 89.0 ± 18.9 | 95.0 ± 18.8 | <0.001 |
| Respiratory rate | 20.2 ± 2.1 | 20.2 ± 1.9 | 20.9 ± 3.4 | <0.001 |
| **Triage** |  |  |  | 0.016 |
| 1 or 2 | 261 (14.6) | 234 (14.0) | 27 (22.0) |  |
| 3 or 4 or 5 | 1529 (85.4) | 1433 (86.0) | 96 (78.0) |  |
| **Symptoms** |  |  |  |  |
| Headache | 102 (5.7) | 97 (5.8) | 5 (4.1) | 0.418 |
| Chest pain | 173 (9.7) | 166 (10.0) | 7 (5.7) | 0.229 |
| Weakness | 141 (7.9) | 128 (7.7) | 13 (10.6) | 0.251 |
| Dyspnea | 160 (8.9) | 140 (8.4) | 20 (16.3) | 0.003 |
| Cough | 129 (7.2) | 110 (6.6) | 19 (15.5) | <0.001 |
| Abdominal pain | 416 (23.2) | 378 (22.7) | 38 (30.9) | 0.037 |
| Vomiting | 218 (12.2) | 199 (11.9) | 19 (15.5) | 0.251 |
| Diarrhea | 93 (5.2) | 81 (4.9) | 12 (9.8) | 0.018 |
| Flank pain | 81 (4.5) | 72 (4.3) | 9 (7.3) | 0.123 |
| Dysuria | 32 (1.8) | 30 (1.8) | 2 (1.6) | 0.889 |
| Urinary frequency | 23 (1.3) | 18 (1.1) | 5 (4.1) | 0.005 |
| Chills | 35 (2.0) | 27 (1.6) | 8 (6.5) | <0.001 |
| Soreness | 85 (4.8) | 74 (4.4) | 11 (8.9) | 0.023 |
| Edema | 77 (4.3) | 69 (4.1) | 8 (6.5) | 0.212 |
| **Lab** |  |  |  |  |
| WBC | 8.9 ± 5.5 | 8.6 ± 3.3 | 11.6 ± 13.1 | <0.001 |
| Seg (%) | 71.8 ± 12.2 | 71.2 ± 12.2 | 76.1 ± 11.5 | <0.001 |
| Hb | 12.7 ± 2.6 | 12.8 ± 2.6 | 12.4 ± 2.5 | 0.153 |
| CRP | 2.2 ± 3.7 | 1.8 ± 3.2 | 3.4 ± 4.7 | 0.006 |
| Na | 134.5 ± 3.7 | 134.6 ± 4.5 | 134.5 ± 4.2 | 0.893 |
| K | 4.0 ± 0.7 | 4.0 ± 0.7 | 3.9 ± 0.7 | 0.736 |
| Cre | 1.6 ± 2.0 | 1.5 ± 2.0 | 1.7 ± 2.2 | 0.534 |
| ALT | 35.5 ± 137.9 | 36.0 ± 145.7 | 32.4 ± 52.9 | 0.815 |
| COPD = chronic obstructive pulmonary disease; CRP = C-reactive protein; DBP = diastolic blood pressure; Hb = hemoglobin; IV_Abx = intravenous antibiotic; SBP = systolic blood pressure; WBC = white blood cell | | | | |
