# Supplementary material for: Intravenous antibiotics at the index emergency department visit as an independent risk factor for hospital admission at the return visit within 72 hours
Source: PLoS One. 2022 Mar 18;17(3):e0264946. doi: 10.1371/journal.pone.0264946 (PMC8932564; doi:10.1371/journal.pone.0264946)
Supplement: S4 Table — (DOCX) [file pone.0264946.s004.docx]

| S4 Table. Comparison of timing of return visit, vitals, symptoms, lab data, diagnosis, and outcome at return visit in the non-infection cohort | | | | |
| --- | --- | --- | --- | --- |
| Variables | Total (n=1,790) | Without IV_Abx (n=1,667) | IV_Abx  (n=123) | *p* |
| **ED returns** |  |  |  | 0.333 |
| <24 h | 803 (44.9) | 751 (45.1) | 52 (42.3) |  |
| 24 h to 48 h | 588 (32.9) | 551 (33.1) | 37 (30.1) |  |
| 48 h to 72 h | 399 (22.3) | 365 (21.9) | 34 (27.6) |  |
| **Vital signs** |  |  |  |  |
| SBP (mmHg) | 146.7 ± 31.5 | 147.1 ± 31.6 | 142.2 ± 29.6 | 0.972 |
| DBP (mmHg) | 79.5 ± 15.9 | 79.7 ± 15.9 | 77.9 ± 16.1 | 0.249 |
| Body temperature | 36.7 ± 0.6 | 36.7 ± 0.6 | 36.8 ± 0.8 | 0.055 |
| Pulse rate (bpm) | 88.4 ± 18.8 | 88.2 ± 18.8 | 91.3 ± 18.4 | 0.083 |
| Respiratory rate | 20.3 ± 2.2 | 20.3 ± 2.2 | 20.6 ± 2.6 | 0.065 |
| **Triage** |  |  |  | 0.154 |
| 1 or 2 | 309 (17.3) | 282 (16.9) | 27 (21.9) |  |
| 3 or 4 or 5 | 1481 (82.7) | 1385 (83.1) | 96 (78.1) |  |
| **Symptoms** |  |  |  |  |
| Headache | 103 (5.8) | 98 (5.9) | 5 (4.1) | 0.405 |
| Chest pain | 155 (8.7) | 147 (8.8) | 8 (6.5) | 0.379 |
| Weakness | 147 (8.2) | 142 (8.5) | 5 (4.1) | 0.083 |
| Dyspnea | 174 (9.7) | 152 (9.1) | 22 (17.9) | 0.002 |
| Cough | 69 (3.9) | 62 (3.7) | 7 (5.7) | 0.273 |
| Abdominal pain | 390 (21.8) | 359 (21.5) | 31 (25.2) | 0.342 |
| Vomiting | 210 (11.7) | 197 (11.8) | 13 (10.6) | 0.678 |
| Diarrhea | 69 (3.9) | 59 (3.5) | 10 (8.1) | 0.011 |
| Flank pain | 76 (4.3) | 69 (4.1) | 7 (5.7) | 0.410 |
| Dysuria | 14 (0.8) | 13 (0.8) | 1 (0.8) | 0.968 |
| Urinary frequency | 7 (0.4) | 6 (0.4) | 1 (0.8) | 0.437 |
| Chills | 19 (1.1) | 17 (1.0) | 2 (1.6) | 0.527 |
| Soreness | 56 (3.1) | 53 (3.2) | 3 (2.4) | 0.649 |
| Edema | 74 (4.1) | 67 (4.0) | 7 (5.7) | 0.369 |
| **Lab** |  |  |  |  |
| WBC | 9.0 ± 4.6 | 9.0 ± 4.5 | 9.7 ± 5.4 | 0.178 |
| Seg (%) | 73.5 ± 11.9 | 73.3 ± 11.9 | 76.0 ± 12.2 | 0.008 |
| Hb | 12.4 ± 2.5 | 12.5 ± 2.5 | 11.9 ± 2.5 | 0.007 |
| CRP | 3.2 ± 5.6 | 2.8 ± 5.0 | 6.7 ± 8.9 | 0.002 |
| Na | 134.2 ± 5.2 | 134.2 ± 5.0 | 134.0 ± 4.5 | 0.961 |
| K | 4.0 ± 1.4 | 4.0 ± 1.4 | 3.8 ± 0.7 | 0.218 |
| Cre | 1.7 ± 2.1 | 1.7 ± 2.2 | 1.4 ± 1.2 | 0.323 |
| ALT | 45.1 ± 159.0 | 44.7 ± 162.3 | 50.5 ± 103.1 | 0.823 |
| **Diagnosis** |  |  |  |  |
| Circulation | 243 (13.6) | 235 (14.1) | 8 (6.5) | 0.017 |
| Respiratory | 139 (7.8) | 123 (9.7) | 49 (13.3) | 0.024 |
| Gastrointestinal | 493 (27.5) | 451 (27.1) | 42 (34.2) | 0.089 |
| **Outcome** |  |  |  |  |
| Hospital admission | 328 (18.3) | 291 (17.5) | 37 (30.1) | <0.001 |
| COPD = chronic obstructive pulmonary disease; CRP = C-reactive protein; DBP = diastolic blood pressure; Hb = hemoglobin; ICU = intensive care unit; IV_Abx = intravenous antibiotic; SBP = systolic blood pressure; WBC = white blood cell | | | | |
