# Supplementary material for: Intravenous antibiotics at the index emergency department visit as an independent risk factor for hospital admission at the return visit within 72 hours
Source: PLoS One. 2022 Mar 18;17(3):e0264946. doi: 10.1371/journal.pone.0264946 (PMC8932564; doi:10.1371/journal.pone.0264946)
Supplement: S5 Table — (DOCX) [file pone.0264946.s005.docx]

| S5 Table. Missing values in vitals | | |
| --- | --- | --- |
|  | Index | Return |
| SBP | 2 | 12 |
| DBP | 2 | 12 |
| Body temperature | 8 | 11 |
| Pulse rate | 2 | 7 |
| Respiratory rate | 1 | 14 |
| DBP=diastolic blood pressure; SBP=systolic blood pressure | | |
